# Students' Feedback Concerning the IMNCI Strategy

**DOI:** 10.4103/0970-0218.45382

**Published:** 2009-01

**Authors:** Mamta Rani, Abhay Kavishvar, Ratan K Srivastava

**Affiliations:** Tutor, Department of Community Medicine, Government Medical College, Surat, India. E-mail: psmmamta@yahool.co.in; 1 Assistant Professor, Department of Community Medicine, Government Medical College, Surat, India; 2 Professor, Department of Community Medicine, Banaras Hindu University, Varanasi, India

Sir,

The government of Gujarat (GOG) has implemented an Integrated Management of Neonatal and Childhood Illness (IMNCI) Strategy under Reproductive and Child Health (RCH) II. A step was taken, perhaps for the first time in India, to sensitize undergraduate (UG) students during their third M.B.B.S. (III/I batch/7th clinical term) by conducting a short training daily during their morning posting to preventive and social medicine for 3 hours over 10 days. Modules and physician chart books were provided to each UG free of cost by the GOG.

In the Department of Community Medicine, Government Medical College, Surat Department of Pediatrics, pre-service IMNCI training for 127 students from batch 78 was conducted jointly in 2007. A self-administered questionnaire containing closed and a few open-ended questions was used to obtain feedback. Although it started with students, it was declared that this training will be a part of the internal assessment.

Out of 127 students, 115 (90.5%) were present when evaluation forms were completed. All of the 115 students attended the training. A total of 91 (79.1%) students attended for all 10 days. Thirteen students (11.3%) were absent for 1 day and the remaining 11 students (9.6%) were absent for 2 days. None of the students were absent for 3 or more days. A total of 60 students (52.2%) found the training to be good; the remaining 55 students (47.8%) thought it was excellent.

Students found the training oriented them towards clinical diagnosis as well as the management of pediatric illnesses. They felt that such training is needed by everyone as it teaches home-based care and is an easy way to reduce morbidity and mortality among children. It provided primary knowledge about child health as well as enhanced their ability to identify serious patients and provide emergency treatment and referral. They valued ward visits and algorithms to classify and manage the pediatric patient.

Training methods were interactive and such practical approaches kept the students attentive. The method of teaching and training and the contents were found by the students to be logical and sequential. Contents were very clear and the method of delivery was lucid. IMNCI training involved mother or parents in treatment, which was beneficial for disease management. A total of 53 students (46.1%) found the training appealing as it used audio-visual aids for demonstration. Fourteen students (12.2%) found the training remarkable because it used disease classification. A majority (72.2%) of the students felt that it improved both their knowledge and skill regarding pediatric practice while 25 of the students (21.7%) thought it improved only their knowledge. The remaining 7 students (6.1%) felt it helped improve only their skill.

A large number of students appreciated all components of the training. They suggested that this training should be given to all categories of health workers throughout India. Two studies that assessed the performance of health workers who had been trained in the full case management process(1,2) showed substantial success in their communication with mothers and in teaching them how to administer treatments at home. One student felt that it was oversimplification for medical students. IMNCI training should be made complex and the time duration should also be increased. Additional visits to wards should be planned and real cases should be given for assessment instead of hypothetical situations.

When asked about the differences between conventional teaching and IMNCI training, students expressed that this is an excellent form of integrated teaching. A different modality of presentations is not seen during the usual teaching of pediatrics and preventive medicine. Active interest was created during this training, which reinforced their existing knowledge.

Most of the opinions from students suggest that it was useful to them but will be more important and meaningful for health workers.

The methodology was impressive and the whole program can increase the confidence of undergraduates in managing common pediatric morbidity and promoting positive behavioral changes.

It will be premature to generalize the observations as it has just been started in Gujarat but students' perceptions and opinions can be of great help in organizing future IMNCI trainings.
